# Patch: platelet transfusion in cerebral haemorrhage: study protocol for a multicentre, randomised, controlled trial

**DOI:** 10.1186/1471-2377-10-19

**Published:** 2010-03-18

**Authors:** Koen de Gans, Rob J de Haan, Charles B Majoie, Maria M Koopman, Anneke Brand, Marcel G Dijkgraaf, Marinus Vermeulen, Yvo B Roos, the PATCH Investigators

**Affiliations:** 1 Department of Neurology, Academic Medical Centre, H2-222, PO-box 22660 1100 DD Amsterdam, The Netherlands; 2 Clinical Research Unit, Academic Medical Centre, PO-box 22660 1100 DD Amsterdam, The Netherlands; 3 Department of Radiology, Academic Medical Centre, PO-box 22660 1100 DD Amsterdam, The Netherlands; 4 Sanquin Blood Bank, North West Region, PO-box 9137, 1006 AC Amsterdam, The Netherlands; 5 Sanquin Blood Bank, South West Region, Department of Research and Development, Leiden, The Netherlands; 6 Department of Immune-Haematology and Transfusion Medicine, Leiden University Medical Centre, Leiden, The Netherlands; 7 Department of Clinical Epidemiology and Biostatistics, Academic Medical Centre, PO-box 22660 1100 DD Amsterdam, The Netherlands

## Abstract

**Background:**

Patients suffering from intracerebral haemorrhage have a poor prognosis, especially if they are using antiplatelet therapy. Currently, no effective acute treatment option for intracerebral haemorrhage exists. Limiting the early growth of intracerebral haemorrhage volume which continues the first hours after admission seems a promising strategy. Because intracerebral haemorrhage patients who are on antiplatelet therapy have been shown to be particularly at risk of early haematoma growth, platelet transfusion may have a beneficial effect.

**Methods/Design:**

The primary objective is to investigate whether platelet transfusion improves outcome in intracerebral haemorrhage patients who are on antiplatelet treatment. The PATCH study is a prospective, randomised, multi-centre study with open treatment and blind endpoint evaluation. Patients will be randomised to receive platelet transfusion within six hours or standard care. The primary endpoint is functional health after three months. The main secondary endpoints are safety of platelet transfusion and the occurrence of haematoma growth. To detect an absolute poor outcome reduction of 20%, a total of 190 patients will be included.

**Discussion:**

To our knowledge this is the first randomised controlled trial of platelet transfusion for an acute haemorrhagic disease.

**Trial registration:**

The Netherlands National Trial Register (NTR1303)

## Background

In studies on outcome prediction after intracerebral haemorrhage (ICH), increasing haematoma volume is a consistent, independent predictor of poor outcome [1,2]. A study with follow-up head CT-scans in the acute phase showed a ≥ 33% increase of haematoma volume during the first 24 hours in at least 38% of patients [3]. This haematoma growth mostly occurs in the first six hours after onset [4,5]. Unsurprisingly, patients suffering from haematoma growth have worse outcome than those without haematoma growth [6]. At our institution we investigated the in hospital mortality after spontaneous ICH. We retrospectively reviewed the charts of all ICH patients who were admitted over a three year period and divided them in three groups. Patients using antiplatelet therapy (APT) had a 40% in hospital mortality (15/38 patients). In patients using oral anticoagulants the mortality rate was 28% during stay in the hospital (11/39) and patients without any antithrombotic medication had a 23% mortality (27/118). This poor outcome for APT patients was unexpected. We first considered this high mortality rate to be an epiphenomenon since the APT patients were older and had more previous cardiovascular events. However, other groups had reported similar results and identified APT use as an independent risk factor for poor outcome [7-12].

A possible explanation for poor outcome in patients on APT is haematoma growth. A population-based study in 208 ICH patients showed that the baseline haematoma volume was comparable between patients who used APT and those who did not [11]. In contrast, patients using oral anticoagulants had a significantly larger haematoma on admission. Although APT use had no influence on haematoma volume on admission, there was more haematoma growth in patients on APT as compared to patients not using any antithrombotic medication. Another study also found that haematoma growth occurs more often in patients using APT [9]. We concluded that antiplatelet agents do not cause a more severe initial haemorrhage, but the hampered platelet function does more often lead to increasing haemorrhage in the first few hours after haemorrhage onset - i.e. haematoma growth.

It seems likely that a treatment strategy which stops this increase of haematoma volume will improve outcome in ICH patients. Such an intervention should be initiated as rapidly as possible to maximise its benefit. It is hypothesised that the best and fastest treatment option to counteract the effect of APT is platelet transfusion. Similar as in other acute life threatening haemorrhagic conditions, such as severe oesophageal bleeding in patients who were using APT, platelet transfusion is often a routine measure. However, a randomised controlled trial to study the effectiveness of platelet transfusion in acute life threatening haemorrhage has never been performed since this treatment is considered to be absolutely necessary or futile.

## Methods/Design

### Objective

The primary aim of the PATCH trial is to investigate whether platelet transfusion reduces poor outcome at 3 months in ICH patients who were using antiplatelet treatment. Poor outcome is defined as a modified Rankin Scale (mRS) score of 4 to 6.

### Study design

The PATCH study is a prospective randomised open label trial with a blind endpoint evaluation (PROBE). This PROBE design is used since placebo treatment for platelet transfusion is not feasible. The development of a placebo is impossible as platelets show a unique swirl in the bag and the option of transfusion using opaque transfusion bags and iv-lines was deemed highly unpractical.

A total of 38 academic, teaching and regional hospitals in The Netherlands are currently participating [appendix I]. Participation of additional centres from the United Kingdom is expected. Other centres are encouraged to participate as well.

### Ethical considerations

Full ethical approval for the study was obtained from the Medical Ethics Committee from the Academic Medical Centre, University of Amsterdam on March 10, 2008. All participating centres' ethical committees assessed the study and provided a declaration of participation.

The PATCH trial will be conducted according to the principles of the Declaration of Helsinki (version of 2004) and in accordance with the Medical Research Involving Human Subjects Act (WMO). Data management, monitoring and reporting of the study will be performed in accordance with the ICH-GCP guidelines.

### Patient population

Patients with ICH who were using antiplatelet agents during the seven days preceding haemorrhage are eligible for the study. This includes patients with ICH on acetylsalicic acid, carbasalate calcium, clopidogrel, dipyridamole or a combination of these drugs. Patients using oral anticoagulants such as warfarin are excluded.

### Inclusion criteria

1. Age ≥ 18 years

2. Non-traumatic, supratentorial ICH confirmed by head CT-scan

3. Glasgow Coma Scale score 8-15

4. Antiplatelet agents used at least during the seven days preceding haemorrhage

5. Treatment can be initiated within 6 hours after onset of first signs and within 1 1/2 hours of the diagnostic CT-scan

6. Pre-stroke modified Rankin Scale score of 0 or 1 (No symptoms, No significant disability despite symptoms; able to carry out all usual duties and activities)

7. Written informed consent is obtained

### Exclusion criteria

1. Haematoma on CT-scan suggestive of epidural, subdural, aneurysmal or arterio-venous

malformation haematoma (judged by treating physicians)

2. Planned surgical evacuation of haematoma within 24 hours after admission

3. Presence of intraventricular blood more than sedimentation in the posterior horns of the lateral ventricles

4. Previous adverse reaction to platelet transfusion

5. Known use of vitamin K antagonists (unless INR ≤ 1.3)

6. Known thrombocytopenia < 100 × 10^9^/l

7. History of coagulopathy

8. Previously legally incompetent adults prior to stroke

9. Death appears imminent

### Outcome

The primary endpoint is functional health at three months scored on the modified Rankin Scale. Poor outcome is defined as a score of 4-6: (moderately) severe disability or death.

Secondary endpoints are: safety of platelet transfusion, the occurrence of haematoma growth, predictive value of the CTA "spot sign" regarding poor outcome and haematoma growth, survival at three months, disability at three months scored using the AMC linear disability score (ALDS), functional health using the full ordinal scoring range of the mRS at three months, poor outcome at three months defined as mRS 3-6, causes of poor outcome and costs.

The primary outcome and a set of secondary outcomes are assessed by a trained research nurse and a radiologist blinded for treatment allocation.

### Sample size

Based on large cohort studies we estimate that in our patient population at least 70% will have poor outcome (mRS 4-6). We aim to reduce this percentage of poor outcome to 50%, a treatment effect which is considered to be highly clinically relevant in ICH. A two group X^2 ^test with a 0,05 two-sided significance level will have 80% power to detect the difference between the control group proportion of 0,70 and an experimental group proportion of 0,50 (odds ratio of 0,429) when the sample size in each group is 95 (total group 190 patients). With this sample size, a two-sided 95% confidence interval for the difference between the proportions will extend 0,136 from the observed difference in proportions.

### Randomisation

Patients are randomised to receive either platelet transfusion or standard care without platelet transfusion. The randomisation procedure is computer- and web-based, using permuted blocks and is stratified by study centre and type of antiplatelet agent (acetylsalicic acid, carbasalate calcium, clopidogrel, dipyridamole or a combination). Randomisation software (ALEA) is made available by the Clinical Research Unit of the Academic Medical Centre.

### Intervention

Platelet transfusion consists of a single gift of 5 or 10 donor units (one or two bags of a standard prophylactic dose, which consists in the Netherlands of 5 pooled buffy-coat derived platelets). The number of donor units depends on the type of medication used. All patients will receive a pooled platelet concentrate of 5 units except for patients using clopidogrel as mono-therapy or in combination with other APT who will receive two pooled concentrates (10 donor units).

The treatment must be initiated as soon as possible but at least within 6 hours after onset of symptoms. This six hour time limit was agreed upon since haematoma growth mainly occurs in the first hours. One of the secondary endpoints is haematoma growth in the first 24 hours. The 1 1/2 hour time window between the diagnostic CT-scan and start of treatment was chosen because of this endpoint. More time between CT and treatment could lead to a failure of treatment effect for this endpoint.

### Data collection

Data will be collected at five distinct moments: on admission, after one hour, after 24 hours (+/- 3), at discharge and after 3 months.

On admission patients characteristics (including age, gender, length, weight, medical history, pre-stroke medication), physical examination (including Glasgow Coma Scale score, stroke severity on the NIH stroke scale), laboratory test results, time between onset of symptoms and treatment start are recorded and the diagnostic CT scan, and a CTA of the cerebral vessels are analysed.

One hour after transfusion platelet count is determined. In selected centres additional platelet function testing is done. After 24 hours a follow-up non-contrast head CT-scan is performed. A third platelet count and platelet function testing is optional. At discharge the date and destination are noted. Follow-up is performed by a telephone interview three months after randomisation.

On the initial and 24 hour CT-scans haematoma volume is measured using computerised volumetric analysis. The spot sign and spot sign score will be scored on the CTA source images. In one centre (St Antonius Hospital Nieuwegein) haematoma volume will be monitored with transcranial doppler (TCD).

### Follow-up measurements

At three months the primary outcome (modified Rankin score) is assessed by a research nurse from the clinical trial bureau of the coordinating centre, who is blinded for treatment, and who will score the functional health outcome by telephone using a structured interview. To increase the inter-observer reliability the number of research nurses will be limited to a maximum of two. An inter-observer variability assessment on primary outcome was performed by the AMC Clinical Research Unit before the PATCH study started. The other clinical outcome measure, the Amsterdam Linear Disability Score (ALDS) will be assessed by the same research nurse during the same telephone interview [13].

The radiological outcomes are assessed by a radiologist blinded for treatment allocation.

### Statistical analysis

Baseline characteristics will be summarized using simple descriptive statistics. The main analysis of the PATCH study consists of a single comparison between the trial treatment groups of the primary outcome after three months (dichotomized mRS score). The analysis will be based on the intention-to-treat principle. The effect size will be expressed in a relative risk estimates and absolute risk reduction. Additionally the primary outcome will be analyzed using multivariate logistic regression, adjusting (if necessary) for clinically relevant baseline imbalances and treatment characteristics. The effect size will be expressed in an adjusted odds ratio (OR). With regard to the range of secondary outcome parameters we will use simple 2 × 2 tables, two-group *t*-tests, Mann-Whitney tests, and multivariate linear and logistic regression models, when appropriate. With respect to the primary outcome predefined subgroup-analyses will be performed: a) different kinds of PAI used by the patient, b) treatment within 2.5 hours versus 2.5 - 6 hours after symptom onset. In all analyses, statistical uncertainty will be quantified via 95% confidence intervals.

### Safety

The study will be carried out in accordance with Dutch legislation and International Conference on Harmonisation/Good Clinical Practice (ICH-GCP) standards. Adverse events are defined as any undesirable experience occurring to a subject during a clinical trial, whether or not considered related to the investigational drug. All adverse events reported spontaneously by the subject or observed by the investigator or his staff will be recorded.

A serious adverse event (SAE) is any untoward medical occurrence or effect that at any dose:

▪ results in death;

▪ is life threatening (at the time of the event);

▪ requires hospitalisation or prolongation of existing inpatients' hospitalisation;

▪ results in persistent or significant disability or incapacity;

If an SAE occurs, the principal investigators will be notified by e-mail or telephone within 24 hours. All SAEs will be reported to the central MEC, according to their requirements.

The investigator will report all suspected unexpected serious adverse reactions (SUSARs) that could have consequences for the safety of the subjects involved in the PATCH-Trial, expedited to the central MEC (Medical Ethics Committee), DSMB (Data Safety Monitoring Board) and competent national authorities, including SUSARs that have arisen in other clinical trials with the same treatment [appendix II].

The DSMB is an independent committee of trial experts who will focus on both safety monitoring and analysis of unblinded effectiveness data. The DSMB consists of three members: 2 clinicians and 1 epidemiologist. The DSMB will perform ongoing safety surveillances, especially with regard to the occurrence of serious adverse events in terms of thrombotic complications of venous or arterial origin and transfusion reactions. The investigator will report the occurrences of these events to the chairman on a weekly basis. The DSMB can recommend the Steering Committee of the PATCH study to early terminate the study when there is clear and substantial evidence of harm [appendix III].

## Discussion

In the PATCH study we aim to improve functional outcome by limiting haematoma growth in ICH patients who are on antiplatelet therapy. The Novoseven phase IIb study, in which ICH patients were randomised to receive recombinant factor VIIa (rFVIIa) or placebo, supported the concept of outcome improvement by hematoma growth reduction [14]. However, in the follow-up phase III FAST trial, rFVIIa had no overall effect on functional outcome despite limiting haematoma growth [15]. A post-hoc subgroup analysis of the phase III trial consisting of 160 patients aged ≤ 70 years, with an ICH volume < 60 ml, an IVH volume < 5 ml and onset to treatment time ≤ 2.5 hours did show a positive effect of rFVIIa on neurological outcome after 3 months [16]. We used the data from this analysis in designing the eligibility criteria for the PATCH study.

Patients with intraventricular extension of the haematoma are excluded. Initially a maximum haematoma size of 60 cc was an inclusion criterion. Many participating centres had difficulty determining haematoma volume and this held back inclusion of patients. Since the other criteria lead to exclusion of patients with a poor prognosis, we decided to withdraw the 60 cc limit. Furthermore, we decided not to include an upper age limit in the inclusion criteria. Instead the patients must have been completely independent before ICH. The 2.5 hour onset-to-treatment time window is very narrow. Although acute stroke patients do arrive in the emergency rooms earlier after onset than before, a window of 2.5 hours would lead to exclusion of many patients. Therefore, we decided to include patients as fast as possible with a maximum of six hours after onset. We intend to perform a subgroup analysis of patients who received the randomised treatment ≤ 2.5 hours after onset.

When designing a study for a treatment that reduces haematoma growth, patient selection is of utmost importance. If a patient's prognosis is already poor, irrespective of treatment - i.e. large infratentorial hematoma or large ventricular extension, reducing haematoma growth cannot improve outcome. Inclusion of such patients will attenuate the possible treatment effect.

The relationship between APT use and outcome has been the subject of considerable debate. A number of studies described prospective cohorts of ICH patients to identify factors that can predict poor outcome in the early stage of the disease. Large haematoma volume, advanced age, decreased state of consciousness and a poor neurological condition are often identified as independent prognosticators. In several cohorts APT use was identified as an independent predictor of poor outcome [7-12]. In other studies the harmful effect of antiplatelets was not established [17-19]. The most recent report by Sansing et al. analysed the placebo treated patients from the CHANT trial [19]. Seventy of 282 patients (25%) were using APT at the time of onset of ICH. The APT patients were older, more likely to be male, had hypertension more often and often had a previous stroke. In this CHANT trial cohort, APT use had no significant effect on haematoma growth or neurological outcome. The authors concluded that prior antiplatelet use does not affect haemorrhage growth or outcome after ICH. However, there are several issues which must be taken into account. The CHANT trial allowed a maximum onset-to-treatment time window of 6 hours [20]. In comparison in the Novo Seven and FAST studies patients were included within 4 hours. Also, the patients published by Sansing had a mean ICH volume at baseline of 21.5 ml. The mean volumes in Novo Seven and FAST were 24 and 23 ml. It has been established that even a small increase in volume has a considerable impact on outcome [6]. The CHANT patients were included later and yet had a smaller haematoma volume. It could be that the patients were a selected subgroup of ICH patient who have a relatively good prognosis regardless of the medication they used. The majority of the patients included in the Sansing study used mono-therapy whereas many patients nowadays receive combinations such as aspirin and dipyridamole. A combination APT regimen might have more effect on hematoma growth and outcome after ICH.

Many colleagues have few doubts on the effectiveness of platelet transfusion in ICH. They argue that a patient with a life threatening haemorrhage who is on APT should have platelet transfusion irrespective of the organ in which the haemorrhage occurred. They feel a trial is not justified since: "we don't have not to prove that water is wet". Others are convinced that platelet transfusion is a waste of money and energy of physicians. Therefore we feel it is justified and important to perform a trial of platelet transfusion in ICH patients with prior APT use.

## Abbreviations

ICH: intracerebral haemorrhage; APT: antiplatelet treatment; GCS: Glasgow Coma Scale; CTA: CT angiography; mRS: modified Rankin Scale; ALDS: AMC linear disability score; WMO: law for medical research with humans; MEC: medical ethics committee; SAE: serious adverse event; SUSAR: suspected unexpected serious adverse reactions; DSMB: data safety monitoring board.

## Competing interests

The authors declare that they have no competing interests.

## Authors' contributions

KdG has made substantial contributions to the conception and design of the study, wrote the first draft of the manuscript and adapted later versions. and has given final approval of the version to be published. RdH has made substantial contributions to conception and design of the study and made critical revisions of the manuscript. CM has made substantial contributions to conception and design of the study and made critical revisions of the manuscript. AB has made substantial contributions to conception and design of the study and made critical revisions of the manuscript. MK has made substantial contributions to conception and design of the study and made critical revisions of the manuscript. MV has made substantial contributions to conception and design of the study and made critical revisions of the manuscript. MD has made substantial contributions to conception and design of the study and made critical revisions of the manuscript. YR conceived of the study, has made substantial contributions to conception and design of the study and made critical revisions of the manuscript. All authors read and approved the final manuscript.

## Appendix I: participating centres with local investigator (s) in alphabetical order

Alysis Ziekenhuis (J. Hofmeijer & S.E. Vermeer), Academisch Medisch Centrum (Y.B. Roos), Atrium Medisch Centrum (C.L. Franke), Canisius-Wilhelmina Ziekenhuis (W.M. Mulleners), Catharina Ziekenhuis (K. Keizer), Elkerliek Ziekenhuis (U.J. Dijkstra), Erasmus Medisch Centrum (F. van Kooten), Flevo Ziekenhuis (I.M. Bronner), Groene Hart Ziekenhuis (L.M.H. Kloos), Jeroen Bosch Ziekenhuis (H.F. Viseé), HAGA Ziekenhuis (S.F.T.M. de Bruijn), Kennemer Gasthuis (H.M.E. Bienfait), Maasstad Ziekenhuis (R. Saxena), Martini Ziekenhuis (J.F. Meilof), Meander Medisch Centrum (T.W.M. Raaijmakers), Medisch Centrum Alkmaar (J.W.M. Brans), Medisch Centrum Haaglanden (K. Jellema), Medisch Centrum Leeuwarden (W.J. Schuiling), Onze Lieve Vrouwe Gasthuis (P. Portegies), Orbis Medisch Centrum (F.A. Rooyer), Reinier de Graaf Gasthuis (L.A.M. Aerden), Rode Kruis Ziekenhuis (W.D.M. van der Meulen), Tergooiziekenhuizen (J.R. de Kruijk), TweeSteden Ziekenhuis (B.P.W. Jansen), Slotervaartziekenhuis (V.I.H. Kwa), Spaarne Ziekenhuis (R.J. Meijer), St. Anna Ziekenhuis (A.E. Boon), St Antonius Ziekenhuis (W.J. Schonewille), St. Elisabeth Ziekenhuis (P.L.M. de Kort), St. Franciscus Gasthuis (S.L.M. Bakker), St. Jansdal Ziekenhuis (D.J. Hofstee), St. Lucas Andreas Ziekenhuis (R.M. van den Berg-Vos), UMC St. Radboud (E.J. van Dijk), UMC Utrecht (C.J.M. Klijn), Vlietland Ziekenhuis (J.C.B. Verhey), VU Medisch Centrum (M.C. Visser), Westfries Gasthuis (T.C. van der Ree).

## Appendix II: data safety monitoring board

- J.B. Reitsma - chairman, Department of Clinical Epidemiology, Biostatistics and Bioinformatics, Academic Medical Centre, Amsterdam, The Netherlands

- C.L. Franke, Department of Neurology, Atrium Medical Centre Parkstad, Heerlen, The Netherlands

- P.W. Kamphuisen, Department of Vascular Medicine, Academic Medical Centre, Amsterdam, The Netherlands

## Appendix III: steering committee members in alphabetical order

L.A.M. Aerden (Reinier de Graaf Gasthuis), R.M. van den Berg-Vos (St. Lucas Andreas Ziekenhuis), H.M.E. Bienfait (Kennemer Gasthuis), K. de Gans (Academisch Medisch Centrum), K. Jellema (Medisch Centrum Haaglanden), E.J. van Dijk (UMC St. Radboud), J.R. de Kruijk (Tergooiziekenhuizen), C.J. Klijn (UMC Utrecht), F van Kooten (Erasmus Medisch Centrum), P.L.M. de Kort (St. Elisabeth Ziekenhuis), J.F. Meilof (Martini Ziekenhuis), T.W.M. Raaijmakers (Meander Medisch Centrum), Y.B.W.E.M. Roos (Academisch Medisch Centrum), R. Saxena (Maasstad Ziekenhuis), W.J. Schonewille (St Antonius Ziekenhuis), J.C.B. Verhey (Vlietland Ziekenhuis), S.M. Zinkstok (Academisch Medisch Centrum).

## Pre-publication history

The pre-publication history for this paper can be accessed here:

http://www.biomedcentral.com/1471-2377/10/19/prepub

## References

[B1] Broderick JP Brott T Duldner JE Tomsick T Huster G Volume of intracerebral hemorrhage. A powerful and easy-to-use predictor of 30-day mortality Stroke 1993 24 987 993 8322400 10.1161/01.str.24.7.987

[B2] Franke CL van Swieten JC Algra A van Gijn J Prognostic factors in patients with intracerebral haematoma J Neurol Neurosurg Psychiatry 1992 55 653 657 10.1136/jnnp.55.8.653 1527534 PMC489199

[B3] Brott T Broderick J Kothari R Barsan W Tomsick T Sauerbeck L Spilker J Duldner J Khoury J Early haemorrhage growth in patients with intracerebral haemorrhage Stroke 1997 28 1 5 8996478 10.1161/01.str.28.1.1

[B4] Fujii Y Takeuchi S Sasaki O Minakawa T Tanaka R Multivariate analysis of predictors of hematoma enlargement in spontaneous intracerebral hemorrhage Stroke 1998 29 1160 1166 9626289 10.1161/01.str.29.6.1160

[B5] Mayer SA Ultra-early hemostatic therapy for intracerebral hemorrhage Stroke 2003 34 224 229 10.1161/01.STR.0000046458.67968.E4 12511778

[B6] Davis SM Broderick JP Hennerici M Brun NC Diringer MN Mayer SA Begtrup K Steiner T for the Recombinant Activated Factor VII Intracerebral Hemorrhage Trial Investigators Hematoma growth is a determinant of mortality and poor outcome after intracerebral hemorrhage Neurology 2006 66 1175 1181 10.1212/01.wnl.0000208408.98482.99 16636233

[B7] Wong KS Mok V Lam WWM Kay R Tang A Chan YL Woo J Aspirin-associated intracerebral hemorrhage: clinical and radiologic features Neurology 2000 54 2298 2301 10881256 10.1212/wnl.54.12.2298

[B8] Roquer J Rodríguez Campello A Gomis M Ois A Puente V Munteis E Previous antiplatelet therapy is an independent predictor of 30-day mortality after spontaneous supratentorial intracerebral hemorrhage J Neurol 2005 252 412 416 10.1007/s00415-005-0659-5 15739042

[B9] Toyoda K Okada Y Minematsu K Kamouchi M Fujimoto S Ibayashi S Inoue T Antiplatelet therapy contributes to acute deterioration of intracerebral hemorrhage Neurology 2005 65 1000 1004 10.1212/01.wnl.0000179178.37713.69 16217049

[B10] Cantalapiedra A Gutierrez O Tortosa JI Yañez M Dueñas M Fernandez Fontecha E Peñarrubia MJ García-Frade LJ Oral anticoagulant treatment: Risk factors involved in 500 intracranial hemorrhages J Thromb Thrombolysis 2006 22 113 120 10.1007/s11239-006-8455-3 17008977

[B11] Saloheimo P Ahonen M Juvela S Pyhtinen J Savolainen E-R Hillbom M Regular aspirin-use preceding the onset of primary intracerebral hemorrhage is an independent predictor for death Stroke 2006 37 129 133 10.1161/01.STR.0000196991.03618.31 16322483

[B12] Lacut K Le Gal G Seizeur R Prat G Mottier D Oger E Antiplatelet drug use preceding the onset of intracerebral hemorrhage is associated with increased mortality Fund Clin Pharmacol 2007 21 327 333 10.1111/j.1472-8206.2007.00488.x 17521302

[B13] Holman R Lindeboom R Vermeulen M Glas CAW de Haan RJ The Amsterdam Linear Disability Score (ALDS) project. The calibration of an item bank to measure functional status using item response theory Quality of Life Newsletter 2001 27 4 5

[B14] Mayer SA Brun NC Begtrup K Broderick J Davis S Diringer MN Skolnick BE Steiner T for the Recombinant Activated Factor VII Intracerebral Hemorrhage Trial Investigators Recombinant activated factor VII for acute intracerebral hemorrhage N Engl J Med 2005 352 777 785 10.1056/NEJMoa042991 15728810

[B15] Mayer SA Brun NC Begtrup K Broderick J Davis S Diringer MN Skolnick BE Steiner T for the FAST trial investigators Efficacy and safety of recombinant activated factor VII for acute intracerebral hemorrhage N Engl J Med 2008 358 2127 37 10.1056/NEJMoa0707534 18480205

[B16] Mayer SA Davis SM Skolnick BE Brun NC Begtrup K Broderick JP Diringer MN Steiner T Can a subset of intracerebral hemorrhage patients benefit from hemostatic therapy with recombinant activated factor VII? Stroke 2009 40 833 840 10.1161/STROKEAHA.108.524470 19150875

[B17] Foerch C Sitzer M Steinmetz H Neumann-Haefelin T for the Arbeitsgruppe Schlaganfall Hessen Pretreatment with antiplatelet agents is not independently associated with unfavorable outcome in intracerebral hemorrhage Stroke 2006 37 2165 2167 10.1161/01.STR.0000231842.32153.74 16809556

[B18] Caso V Paciaroni M Venti M Alberti A Palmerini F Milia P Billeci AMR Silvestrelli G Biagini S Agnelli G Effect of on-admission antiplatelet treatment on patients with cerebral hemorrhage Cerebrovasc Dis 2007 24 215 218 10.1159/000104480 17630480

[B19] Sansing LH Messe SR Cucchiara BL Cohen SN Lyden PD Kasner SE for the CHANT Investigators Prior antiplatelet use does not affect hemorrhage growth or outcome after ICH Neurology 2009 72 1397 1402 10.1212/01.wnl.0000342709.31341.88 19129506 PMC2677505

[B20] Lyden PD Shuaib A Lees KR Davalos A Davis SM Diener H-C Grotta JC Ashwood TJ Hardemark H-G Svensson HH Rodichok L Wasiewski WW Åhlberg G for the CHANT Trial Investigators Safety and tolerability of NXY-059 for acute intracerebral hemorrhage The CHANT Trial Stroke 2007 38 2262 2269 10.1161/STROKEAHA.106.472746 17569876

